# Bioinformatics-based prognostic value and *in vitro* functional validation of PTK6 in cutaneous melanoma

**DOI:** 10.3389/fonc.2025.1555302

**Published:** 2025-07-30

**Authors:** Yanyan Niu, Xiaoyu Liu, Aixiu Shi, Danli Tang, Xiaodong Yao, Yan Lu

**Affiliations:** ^1^ Department of Dermatology, The First Affiliated Hospital of Nanjing Medical University, Nanjing, Jiangsu, China; ^2^ Department of Dermatology, The Affiliated Suqian First People’s Hospital of Nanjing Medical University, Suqian, China; ^3^ Key Laboratory of Neuroregeneration of Jiangsu Province and Ministry of Education, Co-Innovation Center of Neuroregeneration, Nantong University, Nantong, Jiangsu, China; ^4^ Department of Dermatology, The Affiliated Hospital of Nantong University, Nantong, Jiangsu, China

**Keywords:** protein tyrosine kinase 6 (PTK6), cutaneous melanoma (CM), prognostic biomarker, proliferation, invasion

## Abstract

**Background:**

Cutaneous melanoma (CM) is a highly malignant tumor originating from melanocytes. Rising incidence rates pose a significant burden on global health and economy. Advanced CM patients face poor prognosis due to high recurrence and treatment resistance. Identifying new prognostic biomarkers and therapeutic targets is crucial for personalized interventions. This study focused on protein tyrosine kinase 6 (PTK6), whose role in CM remains unclear.

**Methods:**

To overcome these limitations, this study focused on PTK6 and integrated CM transcriptomic and clinical data from TCGA and GEO databases. Bioinformatics analysis evaluated PTK6 expression and its impact on prognosis. GO and KEGG analyses explored biological functions of PTK6-related differentially expressed genes (DEGs). A prognostic risk score model was constructed and validated based on DEGs, and immune cell infiltration, tumor mutation burden (TMB), chemotherapy drug sensitivity, and immunotherapy response were analyzed. Additionally, regulatory mechanisms of PTK6 were explored through mRNA-miRNA-lncRNA and protein interaction networks. Furthermore, *in vitro* experiments validated PTK6's biological functions.

**Results:**

The results showed that PTK6 was significantly upregulated in CM, and its high expression was closely associated with a decreased overall survival of patients. Enrichment analysis suggested that PTK6-related differentially expressed genes were mainly involved in epidermal development, keratinocyte differentiation, and the IL-17 signaling pathway. The prognostic model constructed based on 11 characteristic genes could effectively distinguish between high- and low-risk patients, showing improvements in prognostic accuracy. Patients in the high-risk group had significantly worse prognosis and higher TMB levels. The low-risk group was more sensitive to various chemotherapy drugs, and most immune checkpoint genes were negatively correlated with prognostic genes. TIDE analysis showed that patients in the high-risk group had a higher potential responsiveness to immunotherapy. Regulatory network analysis identified key miRNAs, lncRNAs, and transcription factors related to PTK6. In vitro experiments further confirmed that high expression of PTK6 promoted the proliferation, invasion, and migration of melanoma cells, and its enzymatic active site played an important regulatory role in the above functions.

**Conclusion:**

The experimental results demonstrate that PTK6 is a novel prognostic biomarker and potential therapeutic target for CM, highlighting its strong potential for real-world clinical applications.This study provides a theoretical basis for understanding PTK6's role in CM and its application in personalized treatment. However, further large-scale, multi-center studies are needed to verify its mechanistic role and clinical value.

## 1 Introduction

Melanoma is a malignant tumor that originates from melanocytes, which protect the body from harmful ultraviolet (UV) radiation (1, 2). Melanocytes, derived from the neural ectoderm, migrate to different areas of the body including the skin, eyes, ears, meninges, and gastrointestinal tract. This migration increases the likelihood of melanomas developing in various anatomical sites and organs (1, 3, 4). Melanoma constitutes roughly 1.7% of newly diagnosed primary malignant cancers worldwide and contributes to approximately 0.7% of total cancer-related deaths (5). Among Caucasians, cutaneous melanoma, which represents over 90% of cases, is the prevailing subtype. Conversely, individuals with Black or Brown skin tones and East Asian populations predominantly present with acral melanoma (1, 6, 7). Melanoma, known for its aggressive nature, ranks as the third most prevalent malignant skin cancer after basal cell carcinoma and squamous cell carcinoma (8). Despite its relatively low incidence rate in China, the vast population contributes to approximately 20,000 new cases annually, a number that is steadily increasing, as indicated in the “White Paper on the Current Status of Behavior of Chinese Melanoma Patients.”

In the last decade, the clinical management of melanoma has undergone a revolutionary transformation due to the introduction of BRAF and MEK inhibitors alongside advancements in immunotherapy, leading to a substantial enhancement in the overall survival rates of patients (9). Nevertheless, the prognosis for patients, particularly those with metastatic disease, remains unfavorable due to low response rates and resistance to targeted therapies, resulting in an approximate 27% 5-year survival rate (10). Despite recent advancements in understanding the immunogenomic characteristics of skin melanoma, predictive markers have not been incorporated into clinical practice (11). Cutaneous melanoma, known for its aggressiveness and high metastatic potential, imposes a substantial burden on healthcare providers. Hence, there is an urgent need to identify dependable tumor markers and pathways associated with melanoma progression to facilitate the development of novel therapeutic interventions.

Protein tyrosine kinase 6 (PTK6), also known as breast tumor kinase (BRK), is a cytoplasmic non-receptor protein kinase that functions as an intracellular signal transducer in epithelial tissues. PTK6 was initially identified in cultured human melanocytes in 1993 and subsequently isolated and characterized in breast cancer and mouse intestinal epithelium (12, 13). PTK6, situated on chromosome 20 at positions 13.3-13.4, comprises 8 exons and encodes a 451-amino acid protein. This protein encompasses an SRC homology 3 (SH3) domain, an SRC homology 2 (SH2) domain, and a tyrosine kinase catalytic domain (SH1) (14–17). The SH1 domain, essential for tyrosine kinase activity, is pivotal, and a mutation at lysine residue 219 can lead to loss of kinase activity (18).

PTK6 has been thoroughly investigated in breast cancer, showing elevated expression in different subtypes such as ER-positive, ERBB2-positive, and triple-negative cancers. In a study involving 209 triple-negative breast cancer samples, high PTK6 expression in the lymph node metastasis-positive (LNM+) subgroup was linked to an unfavorable prognosis characterized by reduced overall survival (OS) and disease-free survival (DFS) (19). In human prostate cancer, increased expression of PTK6, its relocation from the nucleus to the cytoplasm, and subsequent activation at the plasma membrane facilitate the phosphorylation and activation of substrates like AKT, p130CAS, and FAK, thereby fostering cancer advancement (20). Chastkofsky et al. showed that PTK6 facilitates UVB-induced skin tumorigenesis in mice by activating signaling molecules including STAT3, FAK, and BCAR1, thereby amplifying inflammatory responses and tumor progression (21). A prior investigation on uveal melanoma proposed that PTK6 potentially enhances the proliferation, migration, and invasion of uveal melanoma cells by suppressing autophagy. Additionally, upregulation of SOCS3 can partially mitigate these impacts, suggesting that modulating the SOCS3-PTK6 signaling pathway could present a viable therapeutic approach for individuals with uveal melanoma (22). PTK6 has been demonstrated to enhance various aspects of tumor progression, such as cell proliferation, migration, invasion, and survival, through the modulation of several signaling pathways, including Ras/MAPK (23), PI3K/Akt/mTOR (24), and JAK-Stat (25). Furthermore, PTK6 plays a role in regulating tumor angiogenesis and apoptosis.

Despite being mentioned in a limited number of studies on melanoma, the association between PTK6 and cutaneous melanoma (CM) has not been investigated. In a prior investigation, we established a prognostic gene signature for CM based on lipid metabolism, comprising 16 genes (including PTK6) identified through ssGSEA, WGCNA, and LASSO Cox regression analyses. Validation through quantitative PCR (qPCR) demonstrated a significant upregulation of PTK6 in CM tumor tissues relative to controls, with elevated expression observed in the high-risk cohort.

In this study, we conducted an in-depth analysis utilizing data from The Cancer Genome Atlas (TCGA) and employing statistical and bioinformatics methodologies to further explore the involvement of PTK6 in the pathogenesis and prognosis of skin cutaneous melanoma (SKCM). Our findings confirm the significance of PTK6 in SKCM development and its potential as a prognostic indicator. Furthermore, experimental validation using melanoma cell lines revealed that PTK6 plays a regulatory role in cell proliferation, migration, and invasiveness. These results collectively position PTK6 as a promising biomarker for both prognostic assessment and therapeutic interventions in SKCM.

## 2 Materials and methods

### 2.1 Data collection

This study predominantly relies on data obtained from the GEO (https://www.ncbi.nlm.nih.gov/geo/) and TCGA (https://portal.gdc.cancer.gov/) databases. The entire genome expression profile and clinical information for SKCM were acquired from the TCGA database using the R package ‘TCGA biolinks (version 2.25.)’ (26). The expression profile and clinical data were obtained from the GEO database through the R package ‘GEO query.’ A total of 473 samples were analyzed, comprising 472 tumor samples and 1 normal control sample from TCGA-SKCM. Additionally, 31 primary melanoma samples from GSE46517 and 46 from GSE15605, each with their respective normal skin control samples, were included in the analysis. Batch effects resulting from non-biological technical variations were adjusted using the ComBat method implemented in the R package “sva” (27). The efficacy of this correction was assessed through principal component analysis (PCA).

### 2.2 Differential expression analysis and prognostic value

The Wilcoxon rank-sum test was employed to compare PTK6 expression between control and tumor groups in the GSE18695 and GSE45917 datasets. To investigate the prognostic significance of PTK6 expression across various cancer types, clinical data from 33 cancer types were retrieved from the TCGA project using the R-package “TCGA biolinks.” A univariate Cox proportional hazards regression model was utilized to assess the association between PTK6 expression levels and patient prognosis. Subsequently, tumors were stratified into high and low PTK6 expression groups based on the median PTK6 levels. Kaplan-Meier survival analysis was then performed to assess the impact of PTK6 expression on overall survival in the aforementioned cancers.

### 2.3 Identification of DEGs and functional enrichment analysis

A total of 549 melanomas were stratified into high and low PTK6 expression groups for analysis. The “limma” package in R was employed to identify differentially expressed genes (DEGs), with samples meeting the criteria of an adjusted false discovery rate |log2Fold Change| >1 and p< 0.05 considered significant. Subsequently, the “clusterProfiler (version 4.2.2)” R package was utilized to conduct Kyoto Encyclopedia of Genes and Genomes (KEGG) pathway and Gene Ontology (GO) analyses based on the identified DEGs.

### 2.4 Establishment and validation of the prognostic signature

Candidate marker genes were assessed through univariate Cox analysis to identify prognosis-related genes (P≤.05). A total of 412 melanoma samples from TCGA-SKCM with accompanying clinical data were randomly split into a training set (n = 282) and a validation set (n = 130) at a ratio of 7:3. The LASSO Cox regression model from the R package “glmnet (28)” was employed to refine the candidate genes and construct a prognostic model. The penalty parameter (λ) was selected based on the minimum criterion. The risk score was computed using the formula:


$$riskScore=\sum_{i=1}^{n}Coef(gene_{i})*Expression(gene_{i})$$

(Coef (genei): coefficient of the gene, Expression (genei): expression level of the gene)

Divided samples into low and high risk groups using the median risk score. Kaplan-Meier curves were plotted for prognostic assessment, and the log-rank test confirmed statistical significance. The ROC curve validated the model’s performance.

### 2.5 Immune infiltration analysis

Single-sample gene set enrichment analysis (ssGSEA) extends Gene Set Enrichment Analysis (GSEA), allowing for the calculation of unique enrichment scores for each sample and gene set. These scores reflect the level of coordinated expression changes of genes within specific gene sets in the sample. Based on the TISIDB (Tumor and Immune System Interactions Database) (http://cis.hku.hk/TISIDB/index.php) database of 28 immune cells, quantifying the relative enrichment score of each immune cell from the gene expression profile of each skin melanoma sample. Differences in immune cell infiltration levels between high and low risk groups were visualized using the R package “ggplot2 (version 3.3.6)”.

### 2.6 Somatic mutation analysis and drug sensitivity analysis

The R package “maftools” is used to display somatic variations between different clusters, including single nucleotide polymorphisms (SNPs), insertions and deletions (INDELs), tumor mutational burden (TMB), and mutation frequency. Based on the half-maximal inhibitory concentration (IC50) data and corresponding gene expression data downloaded from the Genomics of Drug Sensitivity in Cancer (GDSC) database (https://www.cancerrxgene.org/) (29), the potential drug sensitivity of patients with high- and low-risk cutaneous melanoma was predicted using the R package “oncoPredict (version 0.2)” (30).

### 2.7 Immune checkpoint blockade therapy response prediction

We investigated the differences in immune checkpoint expression levels between high and low-risk groups and used TIDE to assess the potential clinical efficacy of immunotherapy in different risk subgroups. The higher the TIDE predictive score, the greater the likelihood of immune evasion, indicating that the patient is less likely to benefit from immune checkpoint inhibitor (ICI) therapy.

### 2.8 Construction of regulatory networks

This study utilized the relationships between target genes provided by the StarBase (https://starbase.sysu.edu.cn/tutorialAPI.php#RBPTarget) online database to construct an RBP-mRNA network. Transcription factors binding to key genes were identified using the hTFtarget database and visualized using the Cytoscape software to form an mRNA-TF interaction network. A ceRNA network was constructed using miRTarBase (https://miRTarBase.cuhk.edu.cn/~miRTarBase/miRTarBase_2022/php/index.php) 、starbase2.0 (https://starbase.sysu.edu.cn/starbase2/index.php) and miRDB (https://mirdb.org/index.html) databases.

### 2.9 Cell lines and culture

The melanoma cell lines A375 (AW-CELLS-H0017) and SK-MEL-28 (AW-CELLS-H0329) were procured from AnWei-sci (Shanghai, China). A375 cells were cultured in Dulbecco’s Modified Eagle Medium (DMEM) supplemented with 10% fetal bovine serum (FBS; Wisent, 086-150), while SK-MEL-28 cells were maintained in Minimum Essential Medium (MEM) supplemented with 10% FBS and 1% penicillin-streptomycin (P/S). These cell lines were incubated at 37°C with 5% CO2. Human epidermal melanocytes (HEMCs) were obtained from Procell Life Science (Wuhan, China) and cultured in Melanocyte Growth Medium under similar conditions.

### 2.10 Western blotting and quantitative real-time PCR

Cellular proteins were extracted utilizing RIPA lysis buffer (Solarbio Life Sciences) and quantified through a BCA assay (Beyotime). Subsequently, 20 µg of protein per sample was resolved on 10% SDS-PAGE gels and transferred onto PVDF membranes for Western blot analysis. Following transfer, membranes were blocked with 5% skim milk in TBST for 1 hour, then probed overnight at 4°C with primary antibodies: anti-GAPDH (1:5000, Proteintech, 60004-1-lg) and anti-PTK6 (1:1000, Abcam, ab233392). HRP-conjugated secondary antibodies, Goat Anti-Mouse IgG(H+L) or Goat Anti-Rabbit IgG(H+L) (Beyotime), were employed. Visualization of protein bands was achieved using ECL reagent (Tanon) and a gel documentation system (Tanon).

Total RNA was extracted using an RNA Extraction Kit (Takara) and reverse-transcribed into cDNA using PrimeScript™ RT Reagent Kit with gDNA Eraser (Takara, RR047A). qRT-PCR was performed with SYBR Premix Ex Taq (Takara, RR820A), and gene expression levels were calculated using the 2−ΔΔCT method relative to GAPDH. The qPCR primers were:

GAPDH-F: 5’-GGAGCGAGATCCCTCCAAAAT-3’;
GAPDH-R: 5’-GGCTGTTGTCATACTTCTCATGG-3’;
PTK6-F: 5’-ACCTGGAGTCGCAGAATTACA-3’;
PTK6-R: 5’-GCCTGGCTAACCCGAAGTC-3’.

### 2.11 Plasmid and small interfering RNA

The human PTK6 coding sequence (CDS) (NM_005975) was cloned into the lentivirus Ubi-3xFlag vector, which was obtained from GeneChem (Shanghai, China). PTK6-specific siRNA sequences were synthesized by GenePharma (Shanghai, China). The sequences are as follows:

siNC: UUCUCCGAACGUGUCACGUtt
siRNA-1: CATCCATGGTTAAGTCATAtt
siRNA-2: CAGGCTGTGCGGCACTACAtt
siRNA-3: AGGTTCACAAATGTGGAGTtt

### 2.12 Cell proliferation assays

A875 and SK-MEL-28 cells (5000 cells/well) were seeded in 96-well plates. Cells were incubated with 10 µL of CCK8 reagent (Beyotime, C0037) at 37°C for 2 hours, following the manufacturer’s protocol. Optical density (OD) was measured at 450 nm using a microplate reader. For colony formation assays, cells were seeded at a density of 200 cells/well in six-well plates and maintained at 37°C with 5% CO2 for 2–3 weeks, with medium changes every 3–4 days. Once visible colonies formed, cultures were terminated, cells were rinsed with PBS, fixed with 4% paraformaldehyde for 15 minutes, and stained with 0.4% crystal violet for 30 minutes. The colonies were then rinsed, air-dried, and counted under white light.

### 2.13 Transwell migration and invasion assay

Cell migration and invasion were assessed using Matrigel-coated transwell chambers (Corning). After transfection, A875 and SK-MEL-28 cells were resuspended in serum-free medium and seeded into the upper chamber at 50,000 cells/well. The lower chamber contained cell culture medium with 10% FBS to stimulate invasion. After 24 hours of incubation, invaded cells were fixed in 4% paraformaldehyde (Aladin, C104188) for 10 minutes and stained with crystal violet (Solarbio, G1070) for 10 minutes. For wound healing assays, transfected A875 and SK-MEL-28 cells were seeded into six-well plates and cultured until 90% confluence. A sterile 200 µL pipette tip was used to create a wound. Cells were then treated with serum-free medium, and wound closure was monitored and photographed at 12 and 24 hours post-scratching.

## 3 Statistical analysis

Bioinformatics analyses were conducted using R software (v4.1.2). Non-parametric data were analyzed using the Wilcoxon rank-sum test, while the chi-square or Fisher’s exact tests were applied for categorical data. Kaplan-Meier survival curves were generated with the “survminer” package, and differences between groups were assessed using log-rank tests. LASSO-Cox regression was employed for gene selection and model building, with the predictive performance evaluated by Receiver Operating Characteristic (ROC) and time-dependent ROC curves. Comparisons between two groups were performed using t-tests, and multiple group comparisons were conducted using one-way analysis of variance (ANOVA) followed by Fisher’s Least Significant Difference (LSD) test.Cellular cellular experiments were performed independently replicated at least three times, and the results are presented as mean ± standard deviation (SD). Statistical significance was defined as a P-value< 0.05.

## 4 Results

### 4.1 Prognostic analysis of PTK6 in pan-cancer

**Figure 1:**
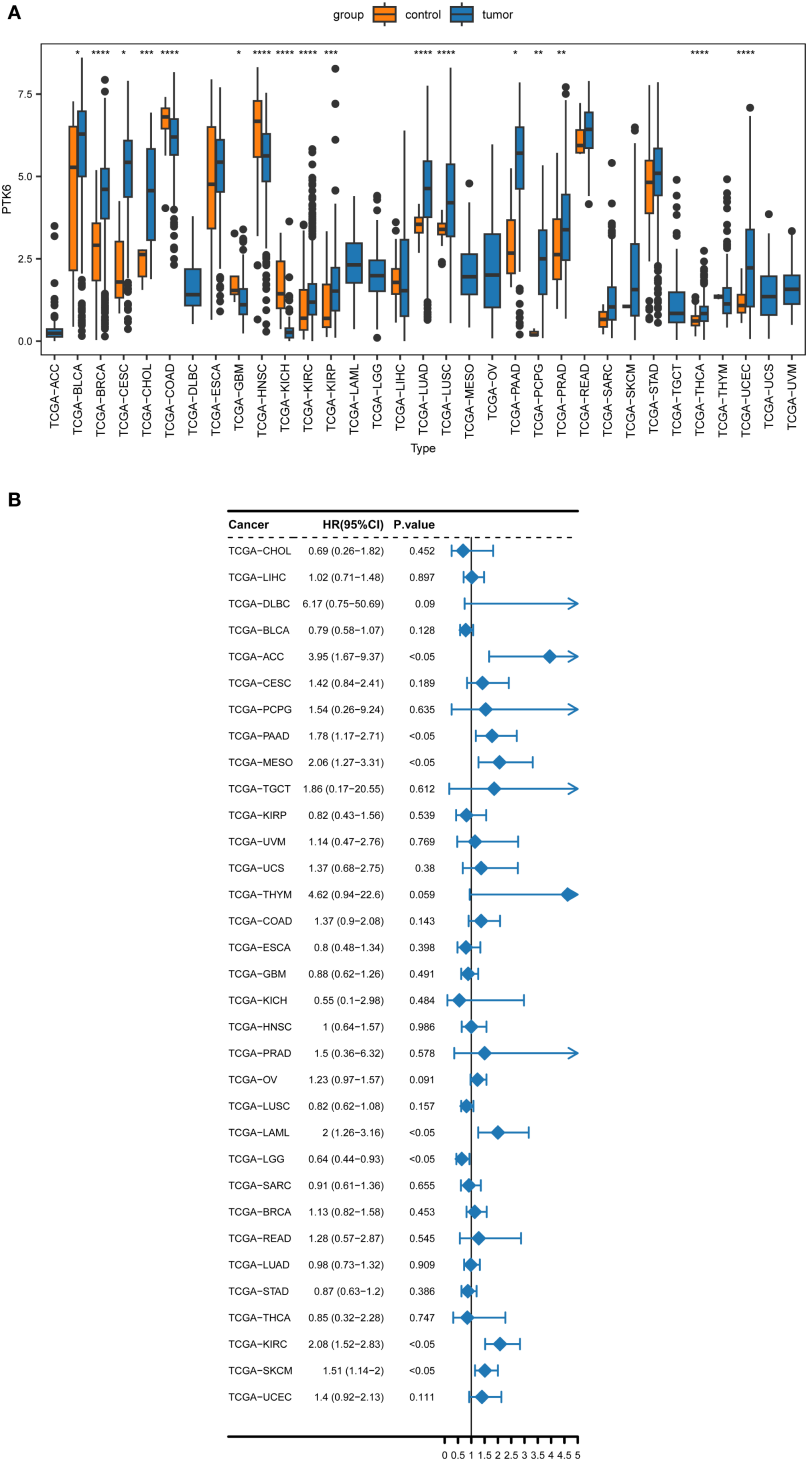
**(A)** The pan-cancer expression analysis of PTK6 in human cancers. **(B)** Forest plot of the univariate Cox regression analysis of PTK6. * denotes P < 0.05, ** P < 0.01, *** P < 0.001, and **** P < 0.0001.

Our prior study identified a notable increase in PTK6 mRNA levels in melanoma samples. To further elucidate the involvement of PTK6 in cancer and SKCM, the present study conducted a Wilcoxon rank-sum test of PTK6 across various cancer types. The results indicated that PTK6 mRNA is significantly upregulated in 17 types of cancers compared to normal tissue samples, including bladder cancer (BLCA), breast cancer (BRCA), cervical squamous cell carcinoma (CESC), cholangiocarcinoma (CHOL), kidney clear cell carcinoma (KIRC), kidney papillary cell carcinoma (KIRP), lung adenocarcinoma (LUAD), lung squamous cell carcinoma (LUSC), pancreatic ductal adenocarcinoma (PAAD), pheochromocytoma and paraganglioma (PCPG), prostate cancer (PRAD), thyroid cancer (THCA), and uterine corpus endometrial carcinoma (UCEC). Conversely, PTK6 expression levels are significantly lower in colon adenocarcinoma (COAD), glioblastoma multiforme (GBM), head and neck squamous cell carcinoma (HNSC), and kidney chromophobe (KICH) when compared to normal tissues (**Figure 1A**). These findings indicate that PTK6 may play specific biological roles and possess clinical significance across various cancer types.

**Figure 2:**
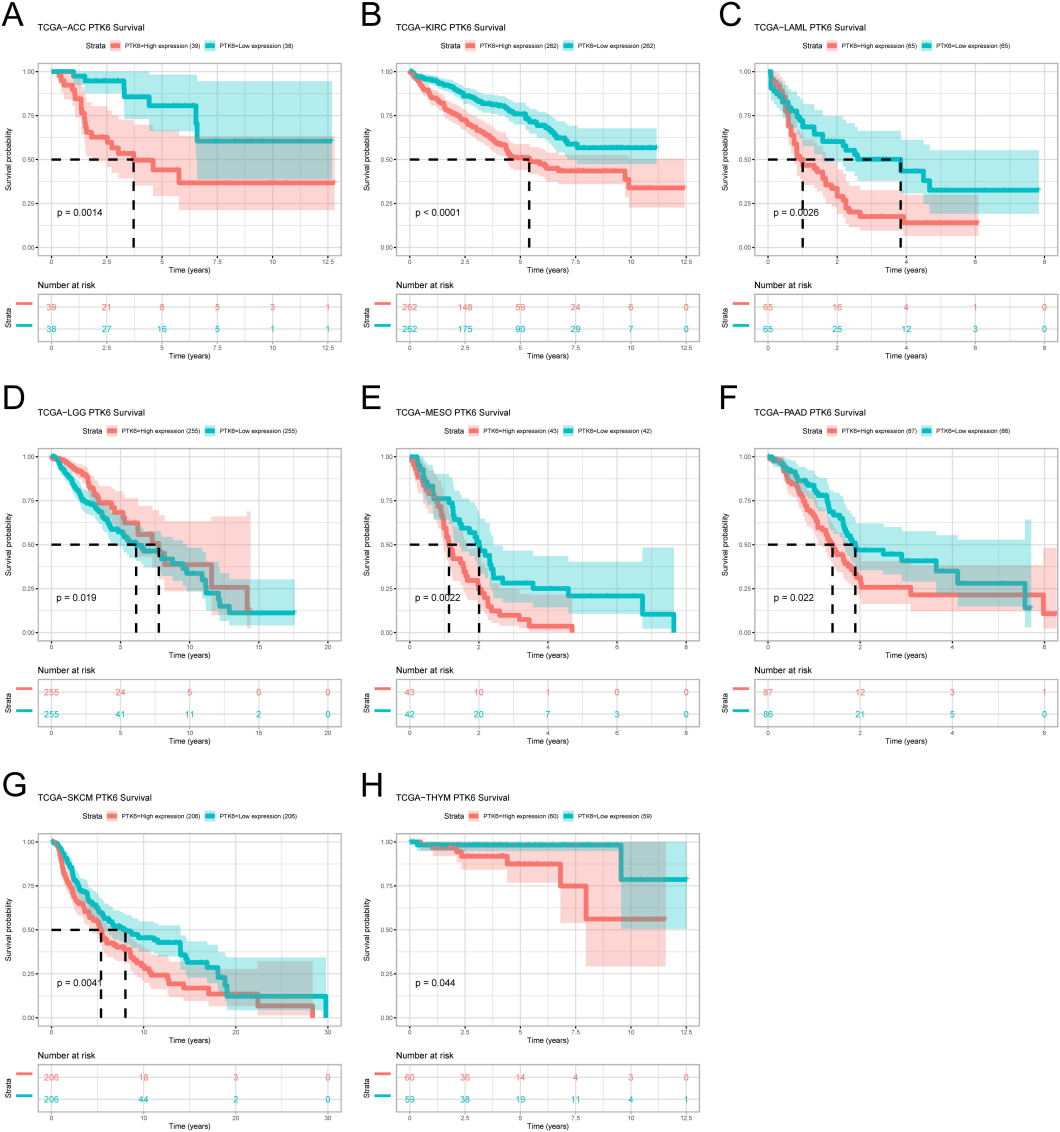
**(A–H)** Kaplan–Meier survival curves estimated overall survival of patients with different expression of PTK6.

To ascertain the prognostic relevance of PTK6 expression across various cancer types, a survival analysis employing the Cox proportional hazards model was conducted. The results revealed a negative correlation between elevated PTK6 expression levels and overall survival (OS) rates. Notably, PTK6 emerged as a detrimental factor in Adrenocortical carcinoma (ACC), Pancreatic adenocarcinoma (PAAD), Mesothelioma (MESO), Acute Myeloid Leukemia (LAML), Kidney renal clear cell carcinoma (KIRC), and Skin Cutaneous Melanoma (SKCM) (**Figure 1B**). These outcomes were subsequently corroborated through Kaplan-Meier survival analysis (**Figures 2A–H**).

### 4.2 Functional analysis of PTK6-related DEGs in SKCM

**Figure 3:**
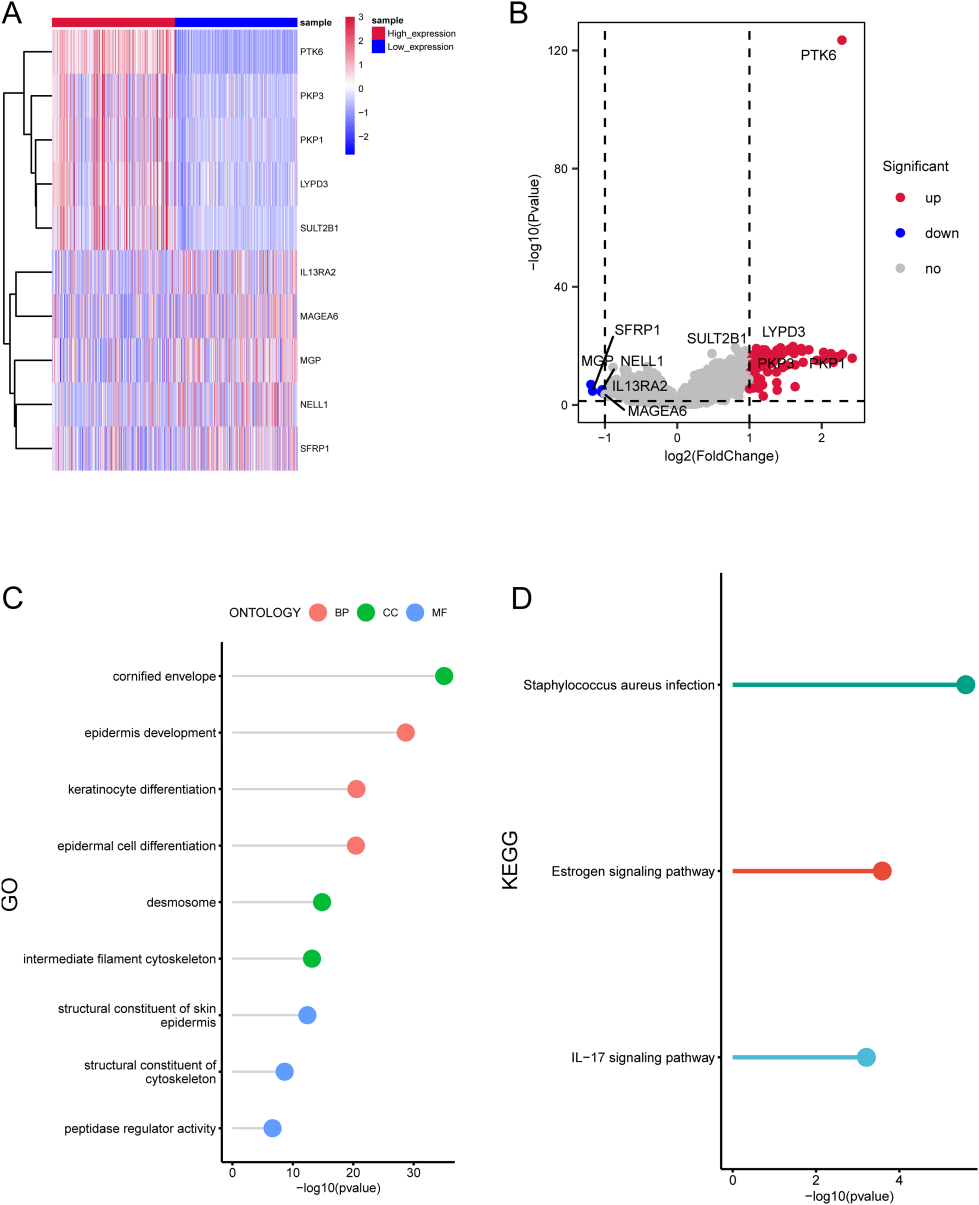
Enrichment analysis of differentially expressed genes related to PTK6 expression. **(A, B)** The top 10 DEGs significantly associated with PTK6 expression in SKCM. **(C)** GO enrichment analysis of the DEGs. **(D)** KEGG enrichment analysis of the DEGs.

To explore additional genes potentially linked to PTK6, we integrated and examined a total of 472 cutaneous melanoma cases from TCGA-SKCM, 31 primary melanoma samples from GSE46517, and 46 primary melanoma samples from GSE15605. These samples were stratified into high (n=275) and low (n=274) expression groups. Our analysis revealed 94 differentially expressed genes (DEGs) associated with PTK6. Among these DEGs, the top 5 upregulated genes (PKP3, PKP1, LYPD3, SULT2B1, LY6D) and the top 5 downregulated genes (IL13RA2, MAGEA6, MGP, NELL1, SFRP1) in the high expression group were visualized using a heatmap (**Figures 3A, B**).

The GO analysis demonstrated the enrichment of these genes in epidermis development, keratinocyte differentiation, epidermal cell differentiation (biological process), cornified envelope, desmosome, intermediate filament cytoskeleton (cellular component), structural constituent of skin epidermis, structural constituent of cytoskeleton, peptidase regulator activity (molecular function) (**Figure 3C**). Additionally, the KEGG analysis affirmed a significant enrichment of these genes in pathways such as peptidase regulator activity, Estrogen signaling pathway, IL−17 signaling pathway (**Figure 3D**).

### 4.3 Construction and validation of prognostic model

**Figure 4:**
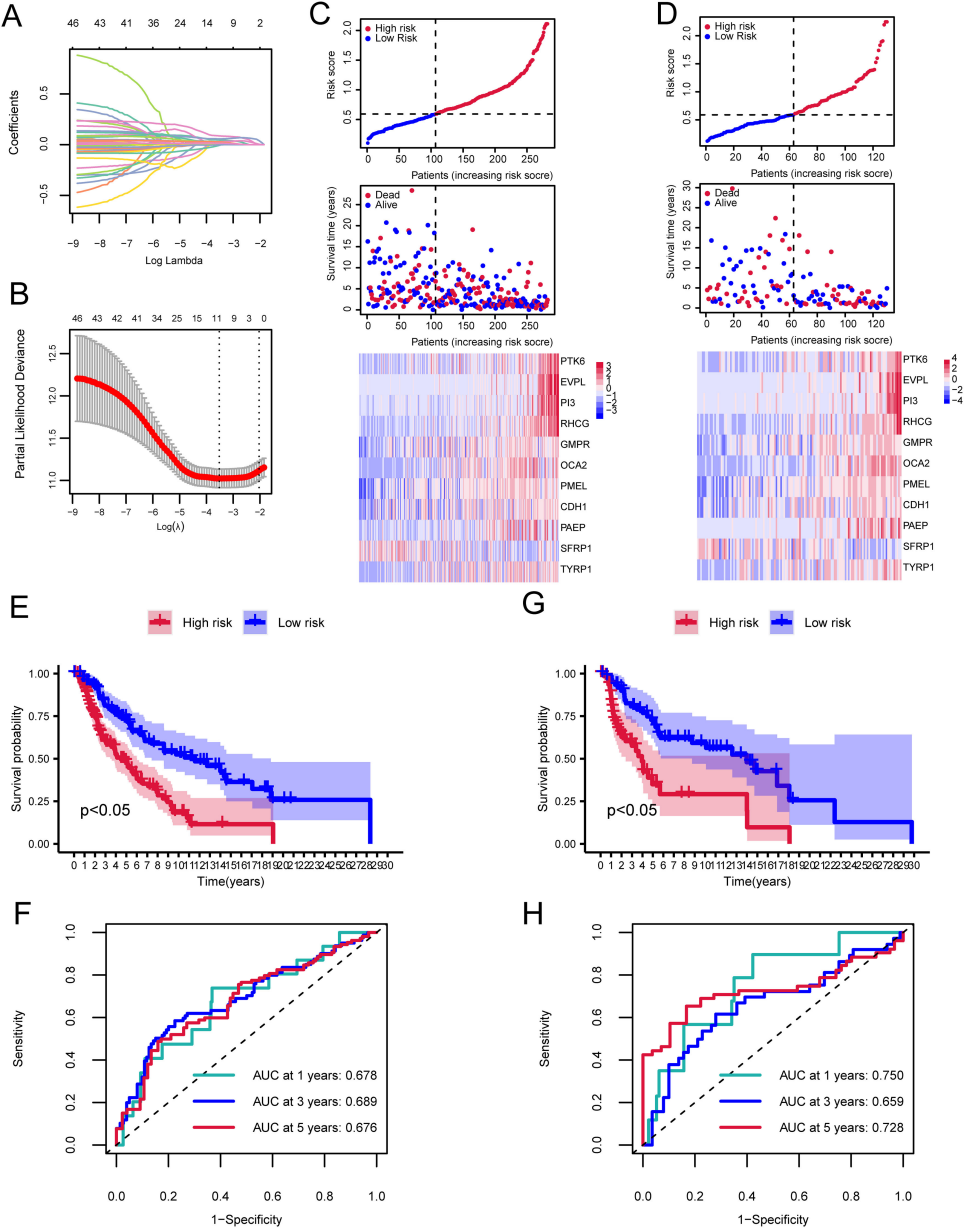
Cox and LASSO regression analyses of the cutaneous melanoma TCGA dataset. **(A)** Trajectories of coefficient changes for predictors in LASSO regression. **(B)** Confidence intervals for each lambda value in the LASSO regression. **(C)** Risk tripartite plot derived from the training cohort. **(D)** Risk tripartite plot derived from the validation cohort. **(E)** Kaplan-Meier survival curves for high-risk and low-risk groups in the training cohort. **(F)** Time-dependent ROC curves for the model in the training cohort. **(G)** Kaplan-Meier survival curves for high-risk and low-risk groups in the validation cohort. **(H)** Time-dependent ROC curves for the model in the validation cohort.

A prognostic model for SKCM was developed using PTK6 expression data from TCGA-SKCM, identifying 47 prognostic-associated genes through univariate Cox analysis of 94 DEGs. A total of 7/10 cutaneous melanoma samples (n=412) were randomly selected to form the training set (n=282), with 3/10 designated as the validation set (n=130). LASSO regression analysis was performed on the training set to eliminate redundant genes. We identified 11 genes (PTK6, EVPL, PI3, RHCG, GMP, ROCA2, PMEL, CDH1, PAEPS, FRP1, TYRP1) linked to the prognosis of cutaneous melanoma patients (**Figures 4A, B**). To evaluate the model’s robustness using this 11-gene signature, samples were categorized into high-risk and low-risk groups based on the median risk score. Risk tertiles (**Figures 4C, D**) and Kaplan-Meier survival curves (**Figures 4E, G**) were generated for patients in both training and validation cohorts. The results showed that patients in the high-risk group had significantly poorer prognosis compared to those in the low-risk group across all cohorts. The ROC curve was used to determine the efficacy of the model in predicting patient prognosis. In the training cohort, the AUC values for 1-year, 3-year, and 5-year survival were 0.678, 0.689, and 0.676, respectively (**Figure 4F**). In the validation cohort, the AUC values for 1-year, 3-year, and 5-year survival were 0.750, 0.659, and 0.728, respectively (**Figure 4H**).

### 4.4 Immune infiltration analysis

**Figure 5:**
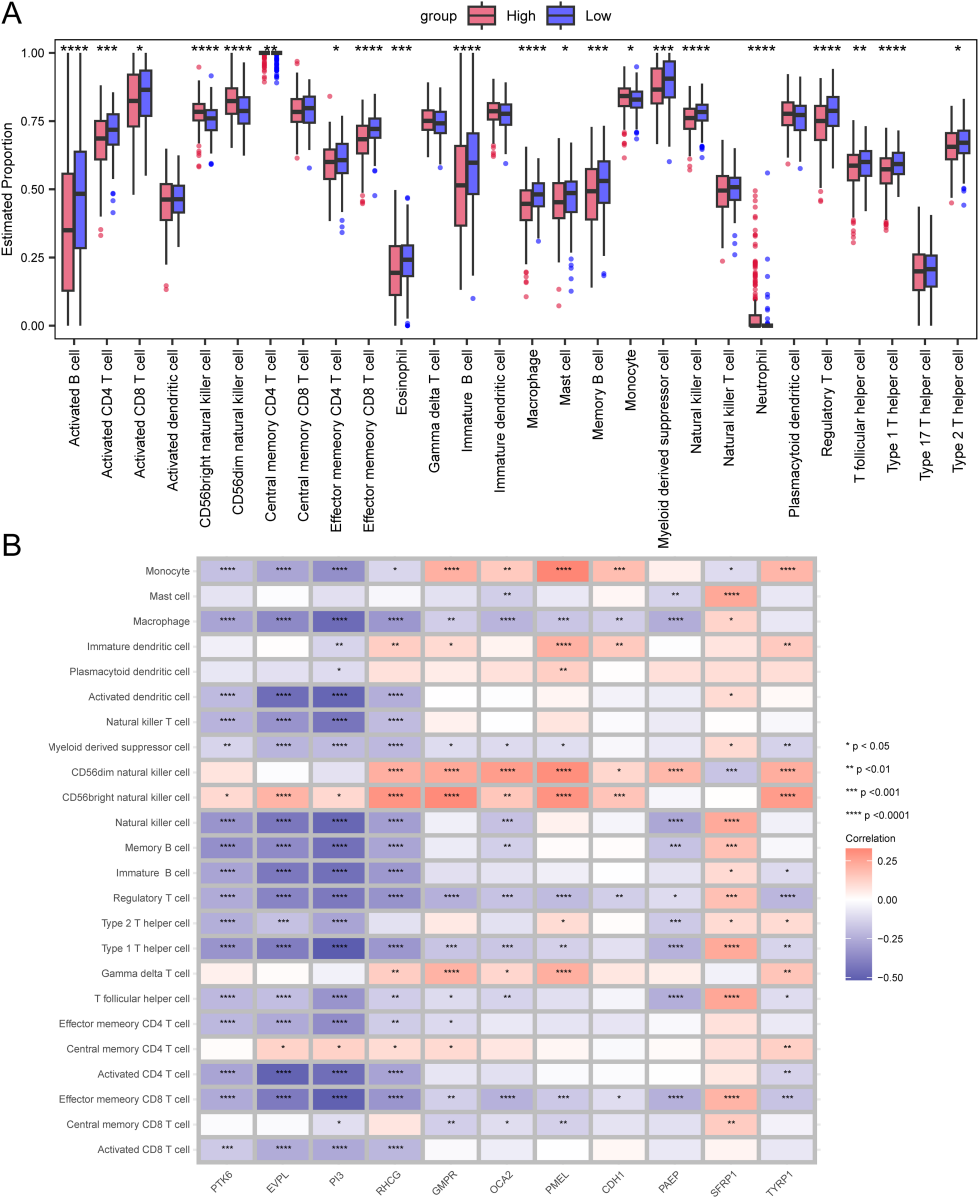
Differences in immune infiltration levels between the high-risk and low-risk groups. **(A)** Box plot representing the estimated proportions of immune cells between high-risk and low-risk groups. **(B)** Correlation between immune cells and prognostic genes. Asterisks indicate P-values: ****p< 0.0001, ***p< 0.001, **p< 0.01, *p< 0.05.

We further investigated the infiltration levels of 28 immune cell types in the high-risk and low-risk groups using the ssGSEA method. Apart from Activated dendritic cell, Central memory CD8 T cell, Gamma delta T cell, Immature dendritic cell, Natural killer T cell, Plasmacytoid dendritic cell, and Type 17 T helper cell, the other immune cells showed significant differences between the high and low-risk groups (p<0.05, **Figure 5A**).

We also demonstrated the correlation between immune cells and prognostic genes through a heatmap. The PTK6 gene is negatively correlated with the vast majority of immune cells and is not significantly correlated with Central memory CD8 T cell, Central memory CD4 T cell, Gamma delta T cell, CD56dim natural killer cell, Plasmacytoid dendritic cell, Immature dendritic cell, and Mast cell, but is positively correlated with CD56 bright natural killer cell (**Figure 5B**).

### 4.5 TMB and drug sensitivity

**Figure 6:**
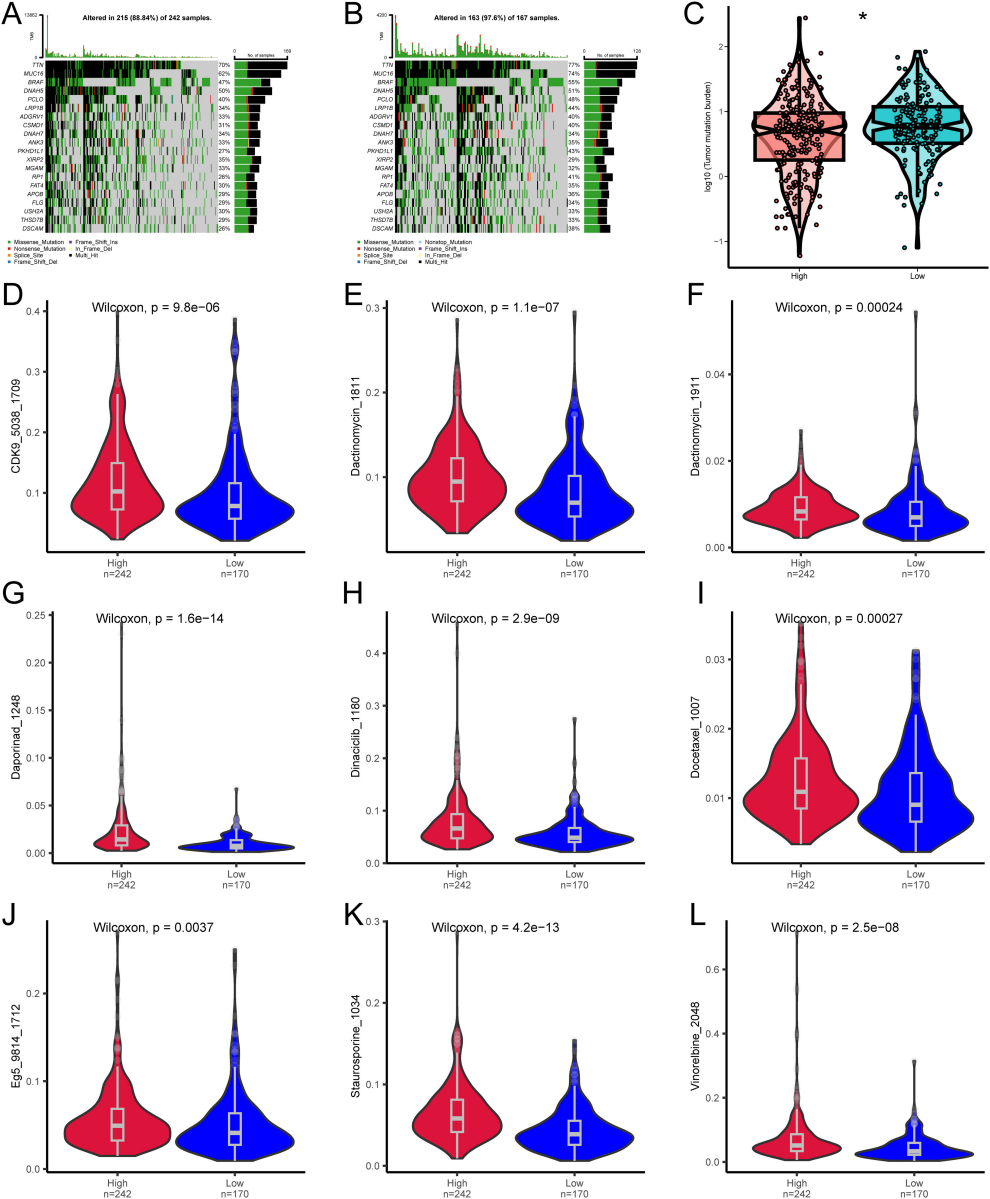
Differences in TMB and drug sensitivity between high-risk and low-risk groups. **(A)** Top 20 most frequently mutated genes in the high-risk group. **(B)** Top 20 most frequently mutated genes in the low-risk group. **(C)** Differences in TMB between high-risk and low-risk groups. **(D–L)** Differences in drug sensitivity between high-risk and low-risk groups. * denotes P < 0.05.

Next, we analyzed the specific gene mutations in melanoma and visualized the top 20 genes with the highest mutation frequency. TTN has the highest mutation frequency in both groups, followed by MUC16 (**Figures 6A, B**). To assess the Tumor mutational burden (TMB), which is a key criterion for evaluating the effects of immunotherapy (24). An analysis of somatic mutations associated with melanoma was conducted, demonstrating that the TMB in the high-risk group was significantly higher than that in the low-risk group (P<0.05) (**Figure 6C**).

We then analyzed whether the risk score can accurately predict the chemosensitivity of melanoma patients. The clinical efficiency of CDK9_5038_1709, Dactinomycin_1811, Dactinomycin_1911, Daporinad_1248, Dinaciclib_1180, Docetaxel_1007, Eg5_9814_1712, Staurosporine_1034, and Vinorelbine_2048 in treating melanoma was studied. The results demonstrated that the patients with a low risk score were more sensitive to the response of CDK9_5038_1709 (**Figure 6D**), Dactinomycin_1811 (**Figure 6E**), Dactinomycin_1911 (**Figure 6F**), Daporinad_1248 (**Figure 6G**), Dinaciclib_1180 (**Figure 6H**), Docetaxel_1007 (**Figure 6I**), Eg5_9814_1712 (**Figure 6J**), Staurosporine_1034 (**Figure 6K**), and Vinorelbine_2048 (**Figure 6L**), indicating that chemotherapy is a promising option for the group with a low risk score.

### 4.6 Immune checkpoint analysis

**Figure 7:**
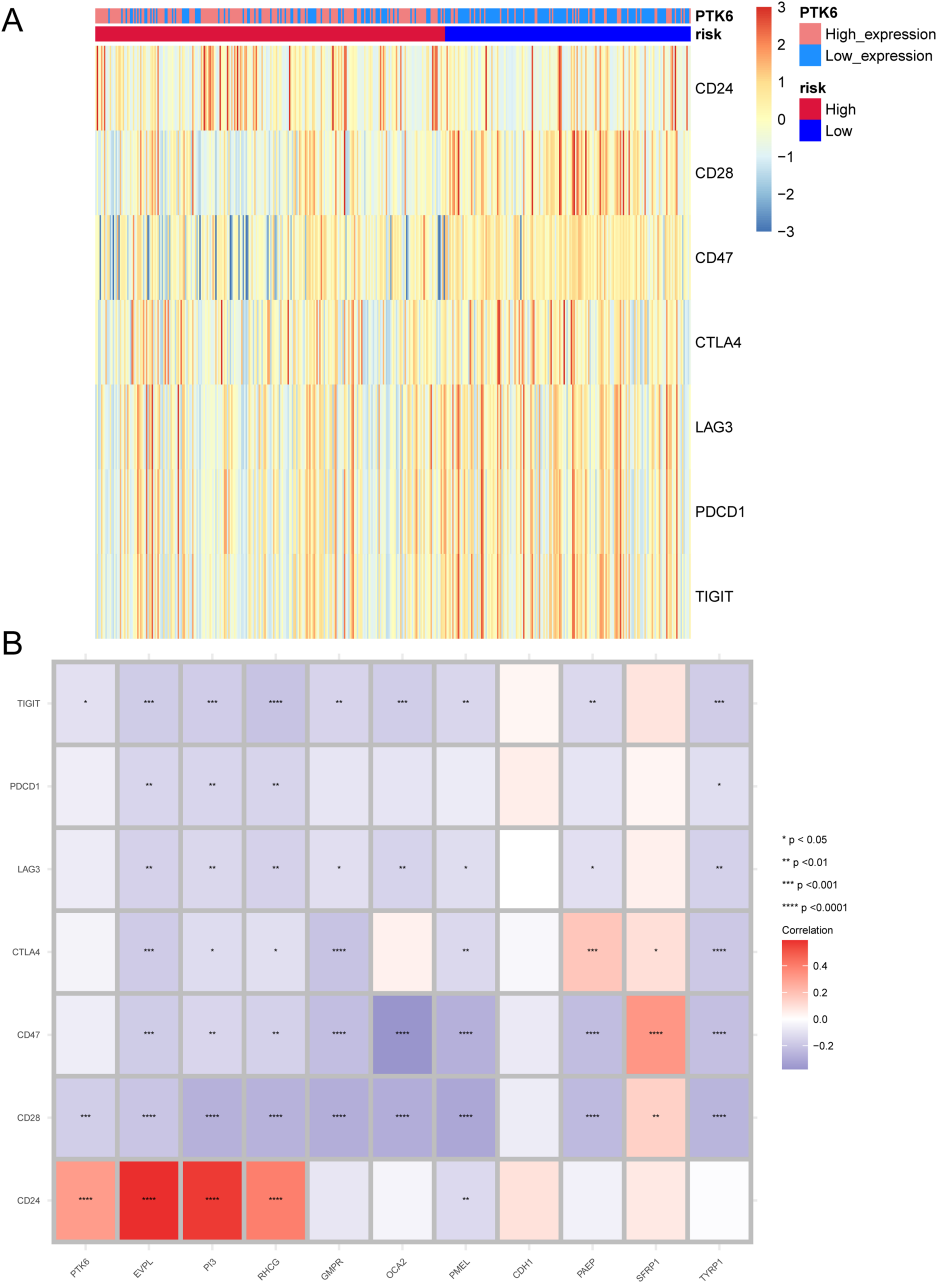
Expression patterns of immune checkpoints. **(A)** Heatmap illustrating the expression of immune checkpoints between high-risk and low-risk groups. **(B)** Correlation between prognostic genes and immune checkpoints. Asterisks indicate P-values: ****p< 0.0001, ***p< 0.001, **p< 0.01, *p< 0.05.

We investigated the differences in the expression levels of immune checkpoint between the high-risk and low-risk groups. CD28, CD47, CTLA4, LAG3, PDCD1, and TIGIT were significantly expressed in the low-risk group (**Figure 7A**). In addition, we also studied the correlation between prognostic genes and immune checkpoints. The expression of vast majority of immune checkpoints are negatively correlated with that of the prognostic genes. The immune checkpoint CD24 is positively correlated with prognostic genes PTK6, EVPL, PI3, and RHCG. Prognostic gene PAEP is significantly positively correlated with the immune checkpoint CTLA4, and prognostic gene SFRP1 is significantly positively correlated with immune checkpoints CTLA4, CD47, and CD28 (**Figure 7B**).

**Figure 8:**
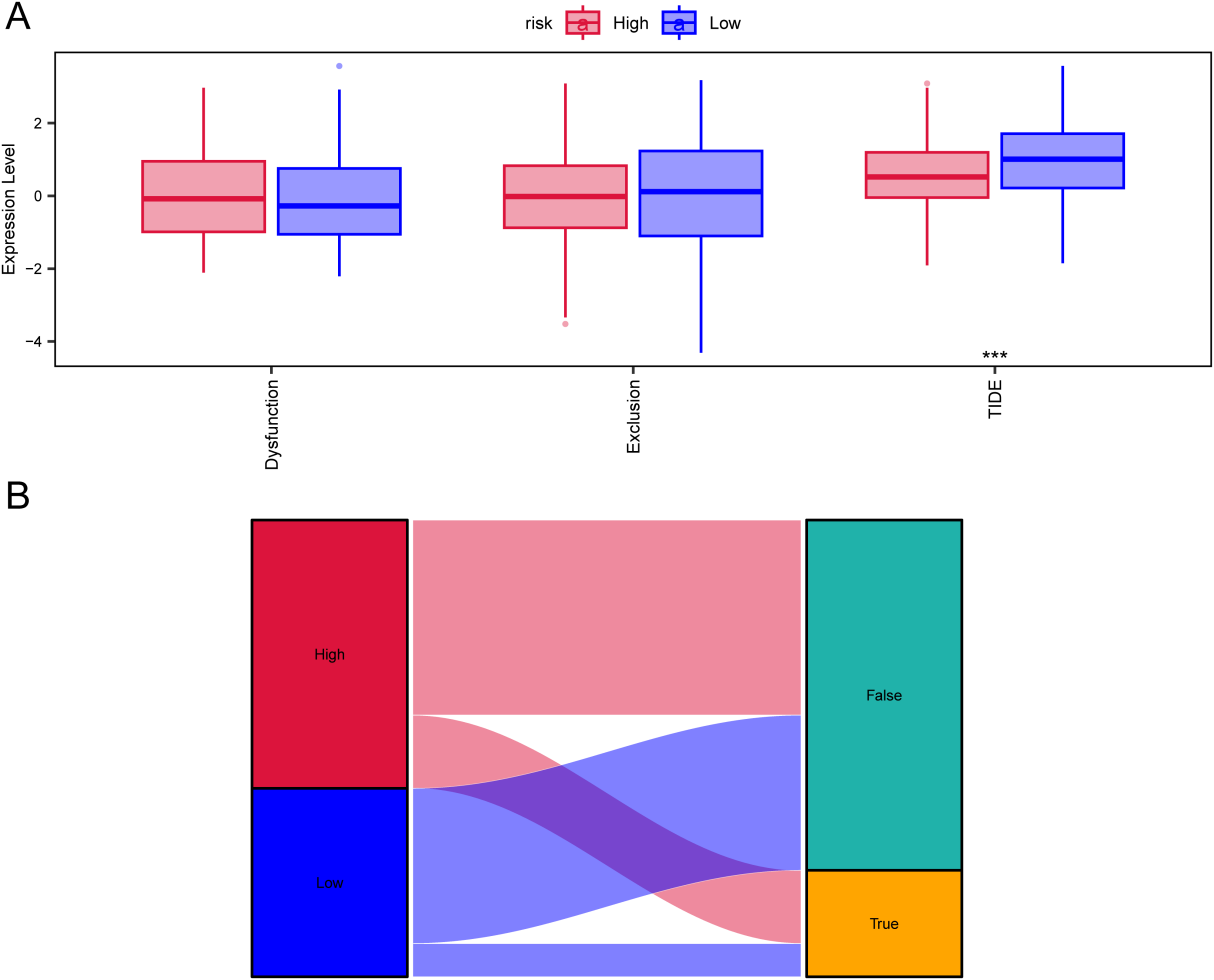
TIDE analysis assessing immunotherapy between high-risk and low-risk groups. **(A)** Differences in T-cell dysfunction, T-cell exclusion, and TIDE scores between high-risk and low-risk groups. **(B)** Sankey diagram illustrating the predicted immunotherapy response based on TIDE analysis.

TIDE is considered to predict the effectiveness of immune checkpoint inhibitors (25).We used TIDE to evaluate the potential clinical efficacy of immunotherapy in different risk subgroups. In the results, the TIDE score of the low-risk subgroup was higher than that of the high-risk subgroup (p<0.05), while there was no significant difference in T cell exclusion and T cell dysfunction between the high and low-risk subgroups (**Figure 8A**). Furthermore, the majority of the responses to immunotherapy came from the high-risk group (**Figure 8B**), which means that patients with higher risk may benefit more from ICI treatment than those with lower risk.

### 4.7 Regulatory network construction

**Figure 9:**
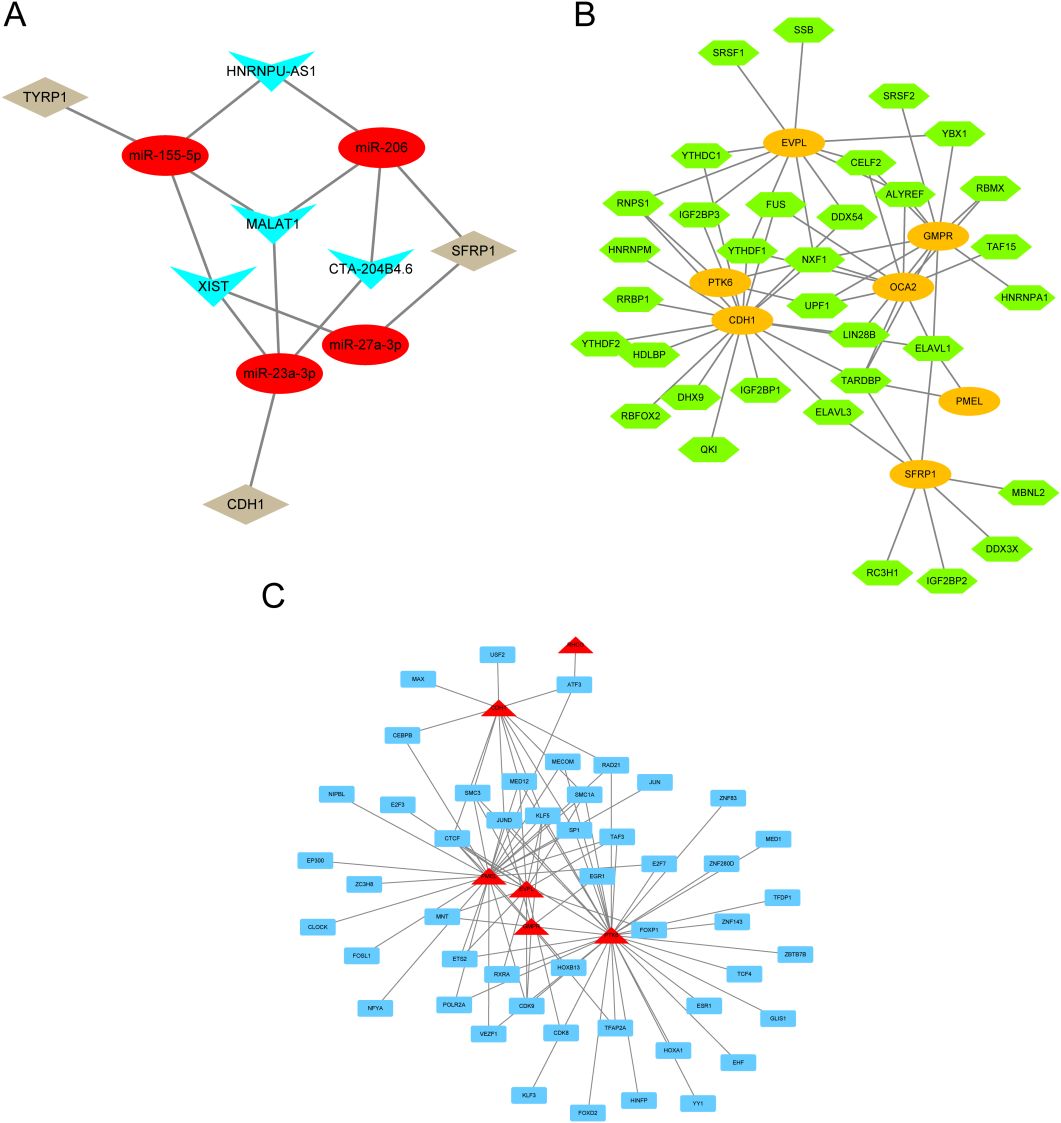
mRNA-miRNA-lncRNA interaction network. **(A)** lncRNA–miRNA–mRNA network for hub genes. Red nodes represent miRNAs, brown nodes represent mRNAs, and blue nodes represent lncRNAs. **(B)** RBP-mRNA regulatory network. Green nodes represent RBPs, and orange circles represent mRNAs. **(C)** mRNA-TF interaction network for hub genes. Red nodes represent mRNAs, and blue nodes represent TFs.

To clarify the underlying molecular mechanisms of prognostic genes in melanoma, we then constructed an mRNA-miRNA-lncRNA interaction network. The data identified miR-155-5p, miR-206, miR-23a-3p, and miR-27a-3p as targeting mRNAs. miR-155-5p binds to TYRP1, miR-206 binds to miR-27a-3p and SFRP1, and miR-23a-3p binds to CDH1. Four lncRNAs, namely lncRNA XIST, lncRNA MALAT1, lncRNA HNRNPU-AS1, and lncRNA CTA-204B4.6, were identified as targets (**Figure 9A**).

Since RBPs (RNA binding proteins) bind to mRNA, we used the StarBase online database to search for and download mRNA/RBP pairs corresponding to 11 prognostic genes, with 7 prognostic genes having corresponding pairing information. Based on the relationships between target genes provided by the online dataset, we constructed an RBP-mRNA network, including 33 nodes, 7 RBPs, 7 mRNAs, and 63 edges (**Figure 9B**).

We searched for transcription factors (TFs) that bind to key genes through the hTFtarget database and ultimately obtained interaction data between 6 prognostic genes (RHCG, PMEL, CDH1, PTK6, EVPL, GMPR) and 49 transcription factors (TFs), which were then visualized using Cytoscape software. In the mRNA-TF interaction network, red triangular blocks represent mRNAs, and blue squares represent transcription factors (TFs) (**Figure 9C**). Among the mRNA-TF interaction network, PTK6 has the most interactions with transcription factors (TFs), with a total of 35 mRNA-TF interaction pairs.

### 4.8 PTK6 is upregulated in SKCM

**Figure 10:**
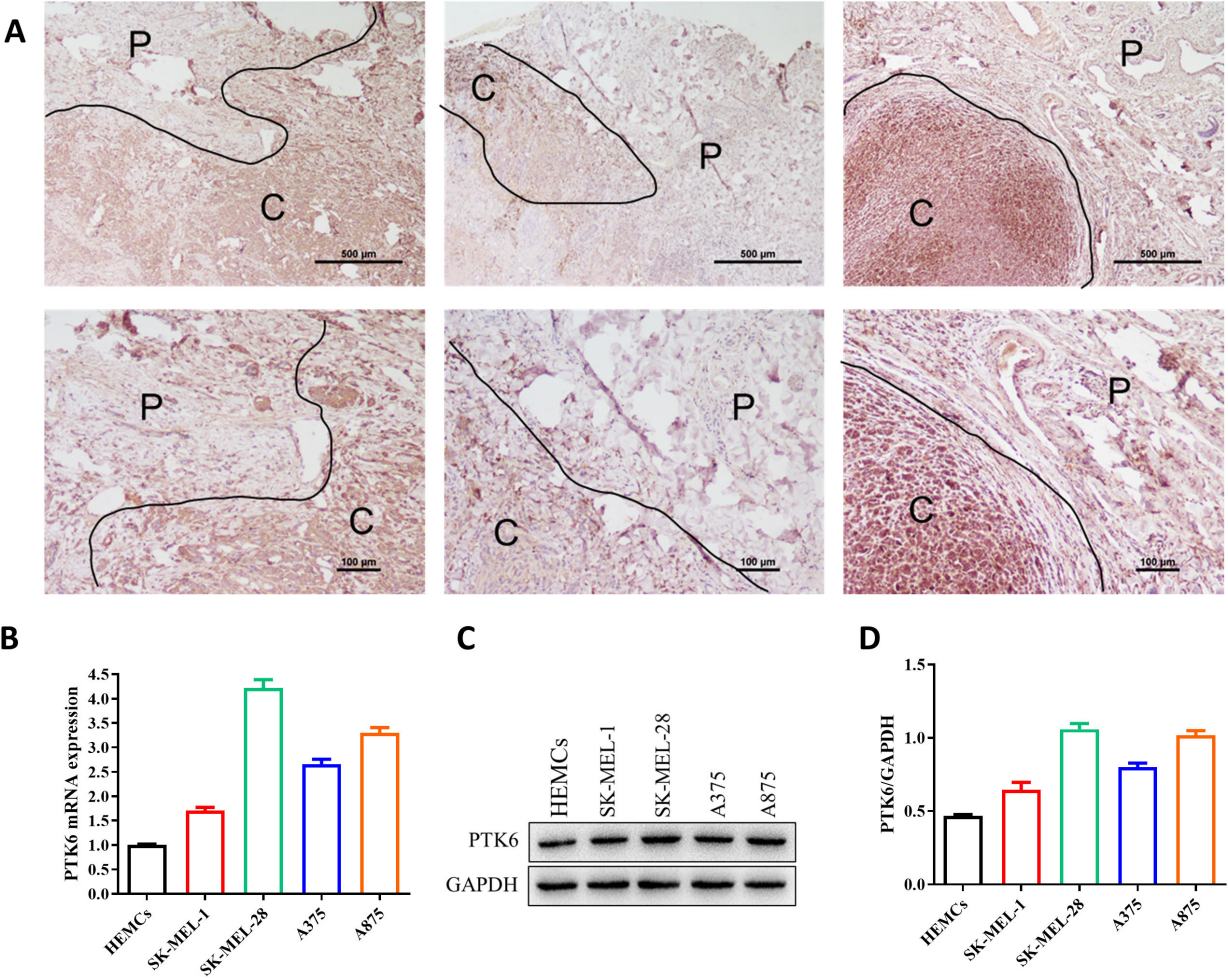
PTK6 is upregulated in SKCM. **(A)** PTK6 expression is higher in cutaneous melanoma tissues compared to adjacent non-tumor tissues. P, Paracancer tissue; C, Cancerous tissue. **(B–D)** Expression of PTK6 in melanoma cell lines and Human epidermal melanocytes (HEMCs).

To further investigate the expression of PTK6 in SKCM, we initially compared the expression levels of PTK6 in tumor tissues and adjacent non-tumor tissues from melanoma patients using immunohistochemistry. The results confirmed that PTK6 expression was significantly higher in tumor tissues compared to adjacent non-tumor tissues (**Figure 10A**), consistent with our previous findings that PTK6 mRNA levels are elevated in tumor tissues. Additionally, using PCR and Western blot (WB) analysis, we observed that PTK6 expression in four melanoma cell lines was markedly higher than in human epidermal melanocytes (HEMCs) (**Figures 10B–D**). These findings collectively indicate that PTK6 is upregulated in cutaneous melanoma, suggesting its potential role as a diagnostic biomarker.

### 4.9 PTK6 promotes SKCM cell proliferation and invasion

**Figure 11:**
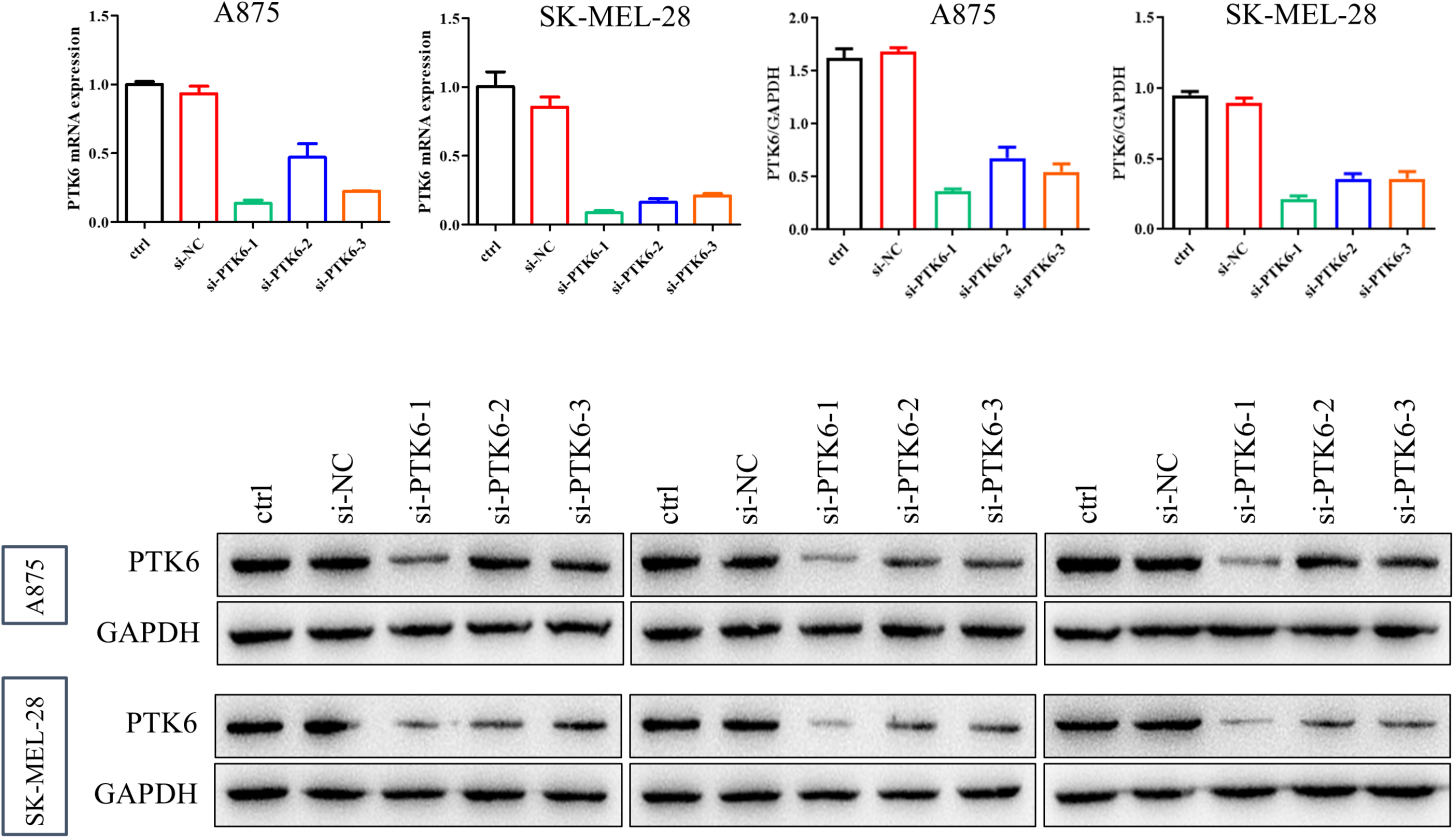
Validation of PTK6 siRNA efficacy.

**Figure 12:**
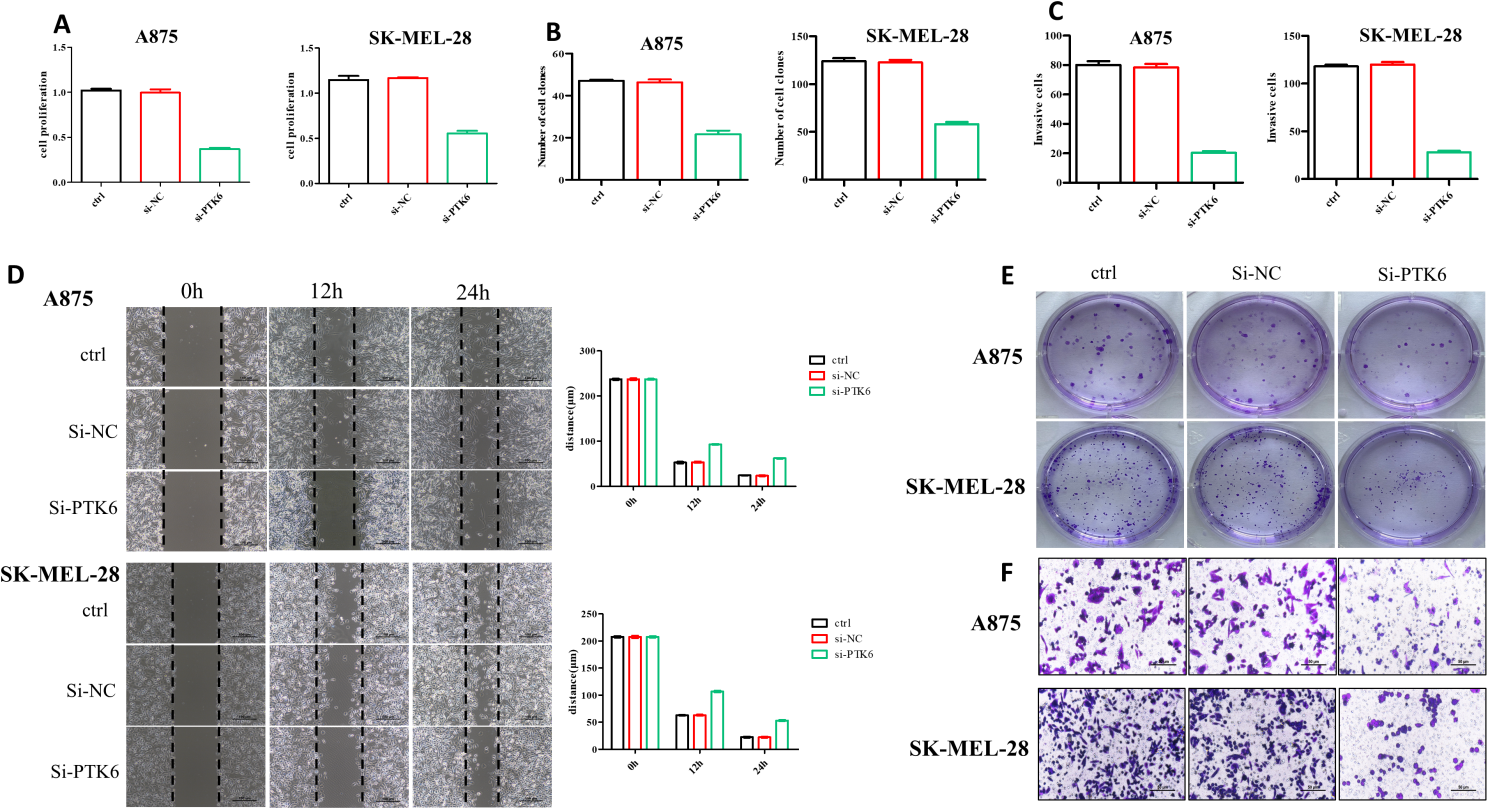
SKCM cells’ proliferation and invasion were diminished in the si-PTK6 group. **(A)** CCK-8 assays showed the proliferation ability of si-PTK6 group was significantly decreased. **(B, E)** Colony formation assays showed cell proliferation in the si-PTK6 group was significantly reduced. **(C, D, F)** Transwell migration assay and wound healing assays demonstrated that the invasion and migration abilities of cells in the si-PTK6 group were markedly lower.

To determine the relationship between elevated PTK6 expression levels and the progression of cutaneous melanoma, we designed the following experiments. First, we constructed si-PTK6 and validated its efficiency in A875 and SK-MEL-28 cell lines using Western blot (WB) and PCR methods. We selected si-PTK6–1 for further experiments as it showed over 60% interference efficiency compared to the si-NC group (**Figure 11**). Subsequently, CCK-8 assays (**Figure 12A**) and colony formation assays (**Figures 12B, E**) revealed that cell proliferation in the si-PTK6 group was significantly reduced in both A875 and SK-MEL-28 cell lines compared to the control (ctrl) and si-NC groups, indicating that PTK6 plays a promotive role in melanoma cell proliferation. Furthermore, wound healing assays (**Figure 12D**) and Transwell migration assay (**Figures 12C, F**) demonstrated that the invasion and migration abilities of cells in the si-PTK6 group were markedly lower than those in the ctrl and si-NC groups, suggesting that PTK6 is functional in promoting invasion and migration mechanisms of melanoma cells. These findings are consistent with bioinformatic analyses, showing the correlation between PTK6 and SKCM prognosis.

### 4.10 K219 site of PTK6 involved in regulating SKCM proliferation and invasion

**Figure 13:**
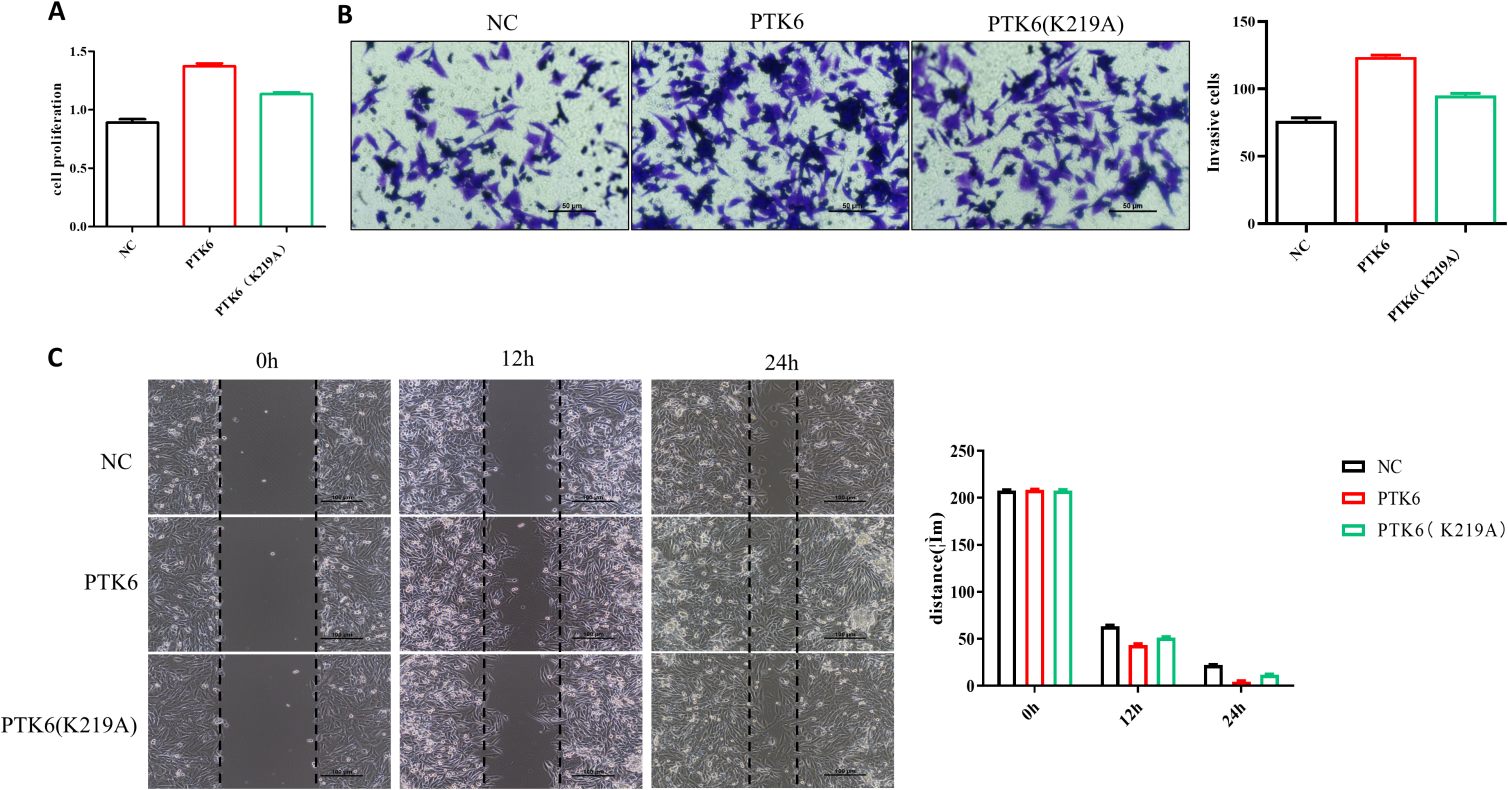
PTK6 (K219A) exhibited decreased proliferation **(A)**, migration **(B)**, and invasion **(C)** capabilities in SK-MEL-28 cells.

PTK6 is a non-receptor tyrosine kinase with classical enzymatic activity sites that are crucial for its catalytic function, including ATP binding sites and substrate binding sites. The K219 site is crucial for catalytic activity, and mutations at this site, such as K219A, typically result in the loss of this activity. We introduced the PTK6 overexpression plasmid and the PTK6 (K219A) mutant plasmid into cells using liposome transfection to achieve respective overexpression of PTK6 and its specific site mutant. The impact of PTK6 enzymatic activity on SKCM proliferation, migration, and invasion was evaluated using CCK-8 assays, Transwell assays, and wound-healing assays. The experimental results showed that, compared to the PTK6-WT group, the PTK6 (K219A) group exhibited decreased proliferation, migration, and invasion capabilities in SKCM (**Figure 13**). This reveals that PTK6 enzymatic activity is involved in these biological processes, providing important evidence for understanding the role of PTK6 in cutaneous melanoma and its potential feasibility as a therapeutic target.

## 5 Discussion

Melanoma, a highly aggressive skin cancer, has a low 5-year survival rate due to its complex etiology and the difficulties in early detection. The rising incidence, especially among young people, poses a significant challenge in managing this tumor. Its aggressive and metastatic characteristics enable swift spread to organs like lymph nodes, liver, lungs, and brain, leading to a poor prognosis (31). While early-stage melanomas can often be cured through surgical removal, metastatic cases may necessitate chemotherapy, radiation, targeted therapies, and immunotherapies (31–34).

Chemotherapy’s effectiveness is limited, and its use is constrained. Although targeted therapy initially appears promising, it encounters high resistance rates. Immunotherapy, particularly immune checkpoint inhibitors (ICIs), has significantly improved survival in advanced melanoma. However, resistance to ICIs emerges in a substantial number of patients, with 60% resistant to monotherapy and 40% to combination therapy (35). Additionally, many patients who initially respond to ICIs may later develop secondary resistance. The prognosis for melanoma remains poor, highlighting the urgent need for more precise biomarker identification and aggressive therapeutic strategies to improve clinical outcomes, especially in BRAF wild-type melanoma (36).

In 1993, Lee ST identified PTK6 in cultured normal human melanocytes (12). Subsequent studies have shown increased expression and activation of PTK6 in various cancers, including breast (19), prostate (37), colorectal (38), and pancreatic cancers (39). A meta-analysis by Jeong et al. assessed the prognostic significance of PTK6 expression in cancer tissues, emphasizing its predictive role across different cancers, with prognostic implications affected by PTK6 levels in adjacent non-cancerous tissues (40).

PTK6 is a critical regulator of key cellular processes, including proliferation, invasion, migration, and epithelial-mesenchymal transition, which collectively contribute to tumor development. It is described as an “effective cooperating oncogene” and is closely associated with the activity of multiple signaling pathways and molecules, such as PI3K/Akt, mTOR, and MAPK, that govern the growth and survival of tumor cells (41). Furthermore, PTK6 can promote the oncogenic signal transduction of various growth factor receptors, including members of the ERBB family, insulin-like growth factor-I (IGF-I), and the epithelial-mesenchymal transition factor (MET) (42, 43).

PTK6 has been linked to tumorigenesis by inhibiting autophagy via the mTOR signaling pathway (22).Bioinformatics analyses have identified autophagy-related genes with prognostic significance in cancers such as breast cancer (44), gastric cancer (45), pancreatic cancer (46), and low-grade glioma (47), using databases like TCGA, GTEx, and GEO. PTK6, an autophagy-related gene, has demonstrated prognostic value across multiple datasets, with its expression correlated with overall patient survival, indicating its potential as a therapeutic target. However, PTK6’s role in cutaneous melanoma pathogenesis remains uninvestigated.

In this study, we performed bioinformatics analysis and *in vitro* validation to investigate the role of the PTK6 gene in SKCM. The Wilcoxon rank-sum test revealed that PTK6 is significantly overexpressed in 17 out of 33 cancer types, notably in bladder, breast, and lung adenocarcinoma, compared to normal tissues. Prognostic analysis using the Cox model showed that elevated PTK6 expression is associated with decreased survival in several cancers, particularly KIRC, SKCM, and PAAD (p<.05). These results were confirmed by Kaplan-Meier analysis and align with previous studies (48, 49). Although PTK6 expression was not significantly elevated in the TCGA dataset, multi-omics analysis, relevant literature, and our qPCR and IHC results demonstrated increased PTK6 expression in SKCM tissues and cells. This discrepancy may stem from inadequate sample selection and limited sample size.

GO and KEGG analyses identified 94 differentially expressed genes (DEGs) associated with PTK6, including 11 genes (PTK6, EVPL, PI3, RHCG, GMP, ROCA2, PMEL, CDH1, PAEPS, FRP1, TYRP1) linked to cutaneous melanoma prognosis. These results align with previous studies. Notably, EVPL inhibits melanoma progression through the RAS/ERK pathway, modifies the tumor microenvironment, and reduces macrophage recruitment (50). High PMEL levels in SKCM correlate with poorer survival and lower infiltration of CD8+ T cells, macrophages, and neutrophils (51). TYRP1 is a target for CAR-T cell therapy in melanoma subtypes unresponsive to immune checkpoint blockade (52). Enriched KEGG pathways include peptidase regulator activity, Estrogen signaling, and IL-17 signaling. This concurs with findings that the TRAF2/PIAS2/ELAVL1/EPHA5 pathway is pivotal in IL-17A-induced melanoma. IL-17A stimulation recruits ELAVL1 and PIAS2 via TRAF2, stabilizing EPHA5 mRNA and promoting melanoma cell proliferation and invasion (53).

We developed a prognostic model based on differential gene expression between high and low PTK6 expression groups in TCGA-SKCM and assessed the infiltration of 28 immune cell types in high- and low-risk groups using the ssGSEA method. Our analysis revealed that patients in the high-risk group had significantly poorer prognoses compared to those in the low-risk group across all cohorts. Except for seven specific immune cells, the high-risk group exhibited higher infiltration levels of various immune cell types than the low-risk group. Correlation analysis between immune cells and prognostic genes showed that PTK6 was negatively correlated with most immune cells but positively correlated with CD56bright natural killer (NK) cells. CD56bright NK cells are highly cytotoxic and can directly target tumor cells. Previous studies indicate that CD56bright NK cells in metastatic lymph nodes (M-LN) are inversely related to melanoma cell percentages and can effectively lyse metastatic melanoma cells upon activation (54). This suggests that CD56bright NK cells are crucial for melanoma immune surveillance. Thus, the positive correlation between PTK6 and CD56bright NK cells may imply that PTK6 affects melanoma progression by modulating NK cell function or activity within the tumor microenvironment.

Patients in the low-risk group showed a lower tumor mutation burden (TMB) than those in the high-risk group and demonstrated heightened sensitivity to several chemotherapeutic agents, such as dacarbazine and docetaxel. This indicates that chemotherapy could serve as an effective, personalized treatment for this subgroup of cutaneous melanoma patients. Our analysis also identified a significant negative correlation between PTK6 expression and multiple immune checkpoint molecules, including PD-1 (PDCD1) and CTLA4. This inverse relationship suggests that tumors with high PTK6 expression may have low expression of key immune checkpoints, thereby affecting the tumor immune microenvironment and modulating the response to immune checkpoint inhibitor therapy.

We observed that while the high-risk group with elevated PTK6 expression had lower immune checkpoint expression, they also showed reduced TIDE scores and a higher likelihood of benefiting from immunotherapy. This suggests that high PTK6 expression may decrease the tumor’s immune evasion by suppressing immune checkpoint molecules, thereby enhancing the tumor microenvironment’s susceptibility to immune clearance during treatment. These results illuminate the complex relationship between PTK6 expression and immune regulation in melanoma, underscoring PTK6’s potential as a predictive biomarker for immunotherapy response. Further studies are needed to elucidate PTK6’s specific role in antitumor immune regulation and the effectiveness of immune checkpoint inhibitors.

To further elucidate the upstream regulatory mechanism of PTK6, this study constructed an mRNA-miRNA-lncRNA interaction network based on multi-database information and systematically analyzed the core regulatory role of PTK6 in the ceRNA network. Through bioinformatics prediction, it was found that miRNAs such as miR- 155-5p, miR-206, miR-23a-3p, and miR-27a-3p could serve as direct regulatory factors of PTK6. Multiple upstream lncRNAs (including MALAT1, XIST, HNRNPU-AS1, CTA-204B4.6, etc.) might indirectly affect the expression level of PTK6 by competitively binding to the above - mentioned miRNAs. Previous studies have confirmed that lncRNAs such as MALAT1 and XIST play a role in promoting proliferation and metastasis in various tumors (55, 56), while miRNAs such as miR-155-5p and miR-206 exert bidirectional regulatory functions of tumor suppression or promotion in the occurrence and development of tumors such as melanoma (57, 58). This ceRNA network suggests that PTK6 is not only directly regulated as a downstream effector molecule of mRNA but may also participate in regulating the proliferation, invasion, and immune environment remodeling of tumor cells through the non-coding RNA network. The above-mentioned mechanism provides new insights into revealing the molecular basis of melanoma progression mediated by PTK6 and also offers a theoretical basis for targeting the PTK6-related ceRNA network to intervene in melanoma.

When analyzing large-sample databases such as TCGA, we found that the somatic mutation frequency of the PTK6 gene was relatively low in cutaneous melanoma, and no obvious high-frequency mutation sites were observed (59). Nevertheless, the expression level of PTK6 and the integrity of its key functional domains are closely related to tumor progression. Existing studies have shown that the enzymatic active sites of PTK6 (such as the K219 site) are crucial for its mediated signal transduction and the biological functions of tumor cells (38, 60). This study further confirmed through functional experiments that mutations at this site would lead to the loss of PTK6 function and significantly inhibit the proliferation and invasion ability of melanoma cells. Therefore, although the overall mutation rate of PTK6 is low in cutaneous melanoma, the integrity of its key domains and expression regulation still have important impacts on tumor biological behavior. In the future, comprehensive studies on the regulatory mutations and genetic variations of PTK6 will help to further elucidate its molecular mechanism and promote its clinical translation as a potential molecular marker and therapeutic target.

No PTK6 inhibitors have yet received clinical approval; however, several potent compounds have emerged from high-throughput screening and computer-aided design. These include natural products, marine compounds, and synthetic chemicals. Phenylmethylene urea derivatives, derived from marine natural products, have shown inhibition of PTK6 phosphorylation in MDA-MB-231 cells and mouse models (61). High-selectivity inhibitors like vemurafenib, PLX472, Tilfrinib, XMU-MP-2, and PF-6683324 effectively target the PTK6 ATP-binding site (62). Furthermore, dasatinib and vemurafenib inhibit PTK6 activity, while oleanolic acid derivatives inhibit PTK6 phosphorylation (63). Current PTK6 drug development emphasizes computer-aided design to identify high-affinity inhibitors, aiming to improve efficacy and minimize side effects (64).

This study confirms PTK6’s involvement in cutaneous melanoma pathogenesis, highlighting its crucial role and therapeutic promise. However, limitations include reliance on public databases for analysis and IC50 data from *in vitro* experiments. Future research should address these limitations by using larger and more diverse sample sets, expanding clinical and animal model experiments, conducting functional studies in additional melanoma cell lines, and exploring PTK6’s therapeutic potential and biological mechanisms. Evaluating PTK6 inhibitors will also be essential for advancing PTK6-targeted therapies. These efforts will provide a stronger theoretical and experimental basis for developing PTK6-targeted therapies.

## 6 Conclusion

Cutaneous malignant melanoma (CM) is a highly aggressive cancer with a growing global incidence and poor prognosis in advanced cases. Despite PTK6’s association with various cancers, its specific role and prognostic significance in CM remain unclear. This study employs a comprehensive approach combining multi-omics bioinformatics analysis and *in vitro* experiments to elucidate the involvement of PTK6 in CM. Our results reveal significant upregulation of PTK6 in CM tissues and cell lines, which correlates with adverse clinical outcomes. By establishing a novel prognostic gene signature, delineating PTK6’s regulatory network, and demonstrating its influence on melanoma cell proliferation and invasion, we identify PTK6 as a promising biomarker and potential therapeutic target for CM. These findings not only advance our current knowledge but also lay the groundwork for future investigations in this field.

## Data Availability

The original contributions presented in the study are included in the article/supplementary material. Further inquiries can be directed to the corresponding authors.
